# Social Network Type and Long-Term Condition Management Support: A Cross-Sectional Study in Six European Countries

**DOI:** 10.1371/journal.pone.0161027

**Published:** 2016-08-18

**Authors:** Ivaylo Vassilev, Anne Rogers, Anne Kennedy, Michel Wensing, Jan Koetsenruijter, Rosanna Orlando, Maria Carmen Portillo, David Culliford

**Affiliations:** 1 NIHR Collaboration for Leadership in Applied Health Research (CLAHRC) Wessex, Health Sciences, University of Southampton, Highfield Campus, Southampton, United Kingdom; 2 University Hospital Heidelberg, Department of General Practice and Health Services Research, Im Neuenheimer Feld, Marsilius Arkaden, Turm West, Heidelberg, Germany; 3 Aarhus University, Department of Public Health, Bartholins Alee 2, 8000, Aarhus C, Denmark; National Institute of Health, ITALY

## Abstract

**Background:**

Network types and characteristics have been linked to the capacity of inter-personal environments to mobilise and share resources. The aim of this paper is to examine personal network types in relation to long-term condition management in order to identify the properties of network types most likely to provide support for those with a long-term condition.

**Method:**

A cross-sectional observational survey of people with type 2 diabetes using interviews and questionnaires was conducted between April and October 2013 in six European countries: Greece, Spain, Bulgaria, Norway, United Kingdom, and Netherlands. 1862 people with predominantly lower socio-economic status were recruited from each country. We used k-means clustering analysis to derive the network types, and one-way analysis of variance and multivariate logistic regression analysis to explore the relationship between network type socio-economic characteristics, self-management monitoring and skills, well-being, and network member work.

**Results:**

Five network types of people with long-term conditions were identified: restricted, minimal family, family, weak ties, and diverse. Restricted network types represented those with the poorest self-management skills and were associated with limited support from social network members. Restricted networks were associated with poor indicators across self-management capacity, network support, and well-being. Diverse networks were associated with more enhanced self-management skills amongst those with a long-term condition and high level of emotional support. It was the three network types which had a large number of network members (diverse, weak ties, and family) where healthcare utilisation was most likely to correspond to existing health needs.

**Discussion:**

Our findings suggest that type of increased social involvement is linked to greater self-management capacity and potentially lower formal health care costs indicating that diverse networks constitute the optimal network type as a policy in terms of the design of LTCM interventions and building support for people with LTCs.

## Introduction

Social network connections have been shown to have a considerable impact on health and well-being outcomes [1–3]. In the arena of long-term condition management (LTCM) a focus on social networks has offered an opportunity to explore the way in which a broad set of contributions from connecting to others and resources can be made available to people in need of LTCM support. Social networks have been identified as a potential vehicle for increasing the effective targeting and promotion of interventions to mobilise and deploy resources and support in LTCM in community and domestic settings [4] and recent evidence suggests that social involvement with a wider variety of people and groups supports personal self-management, emotional and physical well-being. Support work undertaken by personal network members has been shown to have the potential to expand in accordance with health needs assisting individuals to cope with their condition and has the potential to substitute for formal care [5,6].

Existing research evidence implicates the characteristics of networks in promoting or inhibiting the potential of network effects. The amount and nature of illness work undertaken by social network members has been related to increased self-management capacity, improved health, related to quality of life, and reductions in health care utilisation [6,7]. Similarly, network member characteristics (type of relationship, proximity, frequency of contact) have also been found to impact on the amount of illness work undertaken in peoples’ networks and the degree to which support can be substituted for others [5]. There are also suggestions that the degree of substitutability between differently constituted networks, and the level and type of input by different members of a network might change according to circumstances.

Studies focusing on ageing populations have used a combination of network characteristics to illuminate aggregated characteristics from which to construct a set of network types which in turn have produced four main network types (diverse, family, friends, and restricted) that retain national and cross-cultural relevance [8–12]. These network typology studies report that diverse and friend dominated networks were associated with better physical and mental health, morale, and well-being [13], whilst people embedded in less resourceful network types reported lower morale and well-being, and were at greater health risk (e.g. alcohol abuse and physical inactivity) [9,14]. However, there remains a gap in the current literature in terms of identifying the type and associated properties of networks which are likely to optimise LTCM. Thus, our focus in this paper is on exploring types of social networks as a distinctive set of social relationships within which LTCs are managed [14].

In the context of LTCM both value and negative impact has been associated with specific characteristics and properties of networks [15]. Not all networks provide benefits and the key network properties and characteristics (such as the types of relationship, frequency of contact, level of support) are likely to produce differing interactions and influences and operate with a range of contextual influences. For example, studies report different outcomes relative to composition. Vassilev et al. [5] and Koetsenruijter et al. [16] found that higher levels of support from network members may be associated with expanding health need associated with, for example, deteriorating health and negative health behaviours (e.g. smoking). Stoller and Wisniewski [10] report that restricted unsupported networks were associated with more of a sense of well-being than friend supported networks. Network composition and type also vary according to circumstance. For example, Wenger and Scott [17] and Li and Zhang [12] found that deteriorating health leads to withdrawal from more-beneficial diverse networks and shift to less beneficial ones such as family or restricted networks. These differential network effects highlight the relevance of exploring the values, interaction, and properties of networks involved in the specific context of supporting the management of LTCs.

The aim of the analysis presented here is to firstly identify the types of networks relevant for people with LTCs such as diabetes. Secondly to examine the relationship between the type of network that people with diabetes have and their capacity for self-management, sense of well-being, and the amount of support provided by their network members.

## Method

### Study design

Network types have been identified using qualitative [8,18] and quantitative techniques [9]. These studies have generated a range of relevant network categories including type of relationships, frequency of contact, distance from ego, presence of partner and child in the network [8,9]. In this study we follow the quantitative approach developed by Howard Litwin [9], but have adapted the set of variables used in order to reflect relevance for identifying the capacity of different configuration or network types to support LTC management. Our design draws on a cross-sectional study of people with a diagnosis of Type 2 diabetes conducted under the auspices of the European Framework 7 EU-WISE (Whole System Informing Self-management Engagement) project. This project aimed to understand the environmental influences on long-term condition management in order to inform future self-management support (SMS) initiatives [16,19]. The study was conducted between 2011 and 2015 in 6 countries and reflected a variety of health and welfare systems: Greece (GR), Spain (ES), Bulgaria (BG), Norway (NO), United Kingdom (UK), and Netherlands (NL). Respondents were recruited through healthcare practices located in purposively selected areas: deprived urban area; a relatively affluent urban area; and a deprived rural area (relative to country). We planned to recruit 100 patients with a medical diagnosis of type 2 diabetes, and age of 18 years or over, in each area, resulting in about 300 patients from each partner country. Eligible patients were sent an invitation letter with information about the project, a consent form, and a written questionnaire. Patients who completed the questionnaire were invited to take part in a face-to-face interview. Informed consent was obtained in accordance with each country’s ethical guidelines. Written informed consent was given by all patients. Ethical committees in the different countries provided approval for the study; The UNWE and the NCPHA (National Center for Public Health and Analysis) in Bulgaria, the Scientific & Bio-ethical Committee and the Administration Council of the Regional Academic Hospital (PAGNI) of Heraklion in Greece, the CMO region Arnhem Nijmegen in The Netherlands, the Regional Committee for Health and Research Ethics and the ethical committee of the Oslo University Hospital, the Ethics Commission of the University of Navarra, and the University of Manchester Research Ethics Committee, the Greater Manchester Research Ethics Committee, Salford and Trafford local research ethics committee, and the University of Southampton Ethics and Research Governance Online in the UK.

The study used a pre-structured two part patient questionnaire. The first part included validated measures recording demographic variables, quality of life, self-care behaviours, received care and participation in local organizations. The second focused on the mapping of social networks for social support and was conducted face-to-face or over the telephone. The name generator method [20] used probes to generate a list of relevant individuals for family members; friends, neighbours, colleagues; and professional care providers. Next, for each listed individual we collected a number of characteristics, including gender, age, type of connection and the received support according to pre-defined domains: information, treatment, day to day tasks, and emotional support.

### Measures

#### Network typology

In order to construct the typology we included variables used in typologies developed in earlier studies, but adapted them so that they were relevant for LTCM [6,21]. We used the following variables: marital status, pets in the network number of cohabiting children, number of network members, frequency of contact. We calculated frequency of contact as a score of minimum contact days per year for each type of relationship within each network. Each of the answers was given the following scores: at least once a week = 52; at least once a month = 12; at least once every couple of months = 3; at least once a year = 1. The scores were then summed up for each type of network member within each network. For all frequency of contact variables missing values have been interpreted as ‘*no contact*’. The network data was collected face to face (and in a small number of cases over the telephone) and the interviews were done by members of the research team. This means that there was very little missing data that was related to the networks of the respondents.

‘Social network member work’ measures the illness work undertaken by members of a respondent’s network. Participants were asked to first identify the network members relevant for the management of their LTC putting them in three concentric circles depending on their (subjectively assessed) value to the respondent. The interviewees were then asked to assess the contribution of each member of their network in terms of the informational (information related to dealing with one’s illness), emotional (talking about health problems or other personal problems), and practical support (receiving help with practical things in and around the house) on a 1–3 scale (no help, some help, a lot of help) [22]. The scores for each type of work (informational, emotional, practical) within each network were then created as the sum of scores of all network members for each type of support.

#### Self-management of long-term conditions

To measure individual capacity to manage their long-term condition we used the two most relevant subscales ‘*self monitoring and insight*’ and ‘*skills and techniques acquisition*’ of the Health Education and Impact Questionnaire (HEIQ) (http://www.deakin.edu.au/health/research/phi/heiQ.php) [23].

#### Well-being: happiness and mental health

The happiness measure was an item from the European Social Survey (http://www.europeansocialsurvey.org) in which respondents are asked the following question ‘Taking all things together, how happy would you say you are?’ (0–10 scale from extremely unhappy to extremely happy). Subjectively assessed mental health was based on the SF-12v2 questionnaire. SF-12v2 covers eight health domains and includes one or two questions per domain. For the purposes of this analysis summary measures of mental health were calculated following Ware et al. [24].

#### Socio-demographic characteristics

The analysis took into account socio-demographic characteristics that were likely to be relevant for self-management, well-being and network support. This included age, gender, education level achieved, income, employment status and national background based on parental countries of birth.

#### Health status

We used two measures of health status. Subjectively assessed health status was based on the SF12v2 question for general health ‘In general would you say your health is excellent, very good, good, fair, poor’. Respondents were also asked about other conditions in addition to type 2 diabetes and their answers were categorised into ‘no comorbidities’, ‘1or 2’, ‘more than 2’.

### Statistical Analysis

Methods for summarising and analysing data and for estimating associations between outcomes and explanatory variables were chosen based on the suitability of each variable. Continuous variables were assessed for normality and distribution-free tests were used if a substantial departure from normality was observed. Logistic regression was used to assess the effects of explanatory variables on the dichotomised outcome measures in a multivariable adjusted analysis.

The analysis was conducted in three stages. First, we used k-means cluster analysis in order to identify the network types of people with LTCs and explored their sociodemographic characteristics. K-means clustering, originally proposed by Steinhaus in the mid-1950s [25], is a well-established method for splitting a set of data into a given number of groups. It works deterministically, by minimising the Euclidean distance from cluster means, based on a set of variables chosen to discriminate between a pre-set number of clusters. As it is the researchers who select the number of clusters the statistical procedure should be seen as an exploratory one. Following previous Litwin studies [26,27] we experimented with four, five and six cluster solutions. These were the number of clusters obtained in previous studies on social network types conducted in different national contexts. The five cluster solution was chosen by the members of the team that designed the study, and collected and analysed the data, as the most meaningful characterisation of the data, and most reflective of the trends in the literature [26]. Second, we used univariate analytical methods in order to explore the key characteristics of the network types. And finally, we used multivariable regression analysis in order to explore the associations between network types and self-management, well-being, and support work undertaken by network members.

All significance tests were two-sided at the 5% level, with corresponding 95% confidence intervals for all estimates of effect. Statistical analysis was carried out using SPSS version 22 (SPSS software, IBM Corp., Armonk, NY, http://www-01.ibm.com/software/analytics/spss).

## Results

### Stage 1: Typology of personal communities: K-means cluster analysis

Five types of networks were identified by applying the k-means cluster analysis (see Table 1). The following statements about network types derived from the k-means clustering are based on qualitative assessments of relative magnitude of the characterising variables for each network type, and are not based the statistical significance of differences between these groups.

**Table 1:** Network type by delineating characteristics*.

	N	Married	Number of children	Number of network members	Contact with network member	Frequency of contact-Family	Frequency of contact-Friends	Frequency of contact-Other	Frequency of contact-Groups	Frequency of contact-Professionals	Pets
Diverse	88	.67	.31	**4.98**	225	97	**96**	**23**	*17*	8.3	.30
Weak ties	47	.64	.36	3.26	215	152	30	**22**	**116**	**10.6**	.43
Family	145	.77	.51	4.11	**256**	**229**	11	11	*14*	4.3	.37
Minimal family contact	600	.71	.41	2.98	129	109	*9*	*6*	*14*	*5*.*2*	.40
Restricted	982	.62	.37	*1*.*56*	*38*	*29*	*4*	*1*	*20*	*3*.*8*	.35

* Mean values for all variables except Married and Pets where value is proportion with this characteristic.

We performed post-hoc Tukey HSD test on each of the variables used in the typology and the results are included in the table (underlined italics is the subset for lowest values, and bold italics for highest values).

The *diverse* type of network was characterised by numerous and varied network members (family, friends, acquaintances, and groups), who were in more frequent contact with the respondent (‘ego’) than the other types of networks.

The network type entitled *weak ties* was characterised by a diverse set of relationships. However, compared to people in *diverse networks* people in such networks had fewer network members and were less frequently in contact with them. A defining characteristic of the *weak ties network* was of substantive frequent contact with network members who were neither family members nor friends and included voluntary and community groups, health professionals, and acquaintances such as neighbours, colleagues, wardens, taxi drivers.

Two other network types that we identified were family centric dominated by relations with family members but differed in the role these members played: ‘family’ and ‘minimal family’ networks. Respondents clustered in the *family network* were in regular contact with network members and had many network members. However, unlike the other two network types discussed above their contacts were, predominantly with family members, while their engagement with friends and acquaintances was limited.

People with *minimal family* networks maintained most of their contacts with family members and in this respect their networks were similar to the *family network*. However, unlike *family networks* they possessed fewer network members and were not generally in frequent contact with them. Additionally, the contact that people with *minimal family support* networks had with non-family network members was minimal.

The *‘restricted’* network was characterised by few social network members and contact time with network members of all kinds was low. People in such networks were likely to be isolated with contacts likely to be limited to contact with a partner and/or a live-in child.

### Stage 2: Network types: socioeconomic characteristics, self-management, well-being, and work undertaken by personal community members

The second stage of analysis focused on exploring the underlying characteristics of each of the network types (see Table 2). Most of the respondents in our sample were in *restricted* and *minimal family* networks, which was also consistent across all the countries in the study. The two diverse network types, *diverse* and *weak ties*, constituted less than 8% of the total. There were only a small number of people with *diverse* and *weak ties* networks in Spain, while these networks were almost entirely absent in Greece. Norway and UK had the highest proportion of *diverse* and *weak ties* networks.

**Table 2:** Characteristics of social network types: cross tabulations and one-way analysis of variance.

		Network type				
	Diverse	Weak ties	Family	Minimal family	Restricted	Statistic
**Socioeconomic characteristics**						
***Gender***						
Men, n(%)	48(54.5)	23(50)	56(39.4)	277(46.6)	523(54.1)	
Women, n(%)	40(45.5)	23(50)	86(60.6)	318(53.4)	443(45.9)	X^2^ = 16.36**
***Family background***						
Both parents native, n(%)	76(86.4)	40(87.0)	126(88.1)	552(93.9)	852(88.5)	
One or both parents born abroad, n(%)	12(13.6)	6(13.0)	17(11.9)	36(6.1)	111(11.5)	X^2^ = 14.70**
***Country***						
*Bulgaria*, n(%)	20(22.7)	4(8.5)	13(9.0)	128(21.3)	135(13.7)	
*Greece*, n(%)	1(1.1)	0(0)	20(13.8)	139(23.2)	145(14.8)	
*Netherlands*, n(%)	8(9.1)	20(42.6)	37(25.5)	65(10.8)	174(17.7)	
*Norway*, n(%)	30(34.1)	9(19.1)	17(11.7)	97(16.2)	147(15.0)	
*Spain*, n(%)	5(5.7)	3(6.4)	37(25.5)	84(14.0)	171(17.4)	
*United Kingdom*, n(%)	24(27.3)	11(23.4)	21(14.5)	87(14.5)	210(21.4)	X^2^ = 158.20***
***Number of network members***, M(SD)	4.98(2.6)	3.26(2.6)	4.11(2.5)	2.98(1.4)	1.56(1.5)	K = 532.01***
***Education***						
No education, n(%)	0(0)	1(2.1)	7(5.0)	19(3.2)	30(3.1)	
Primary school, n(%)	6(6.8)	7(14.9)	48(34.0)	183(30.9)	252(26.1)	
Secondary school (up to 16 years), n(%)	31(35.2)	19(40.4)	31(22.0)	169(28.5)	313(32.4)	
College, n(%)	30(34.1)	13(27.7)	35(24.8)	140(23.6)	228(23.6)	
University, n(%)	21(23.9)	7(14.9)	20(14.2)	81(13.7)	144(14.9)	X^2^ = 43.23***
***Employment status***						
Not employed, n(%)	51(59.3)	38(82.6)	110(80.9)	464(78.9)	738(77.1)	
Employed part/full time, n(%)	35(40.7)	8(17.4)	26(19.1)	124(21.1)	219(22.9)	X^2^ = 18.48**
***Income***						
Lower than average, n(%)	48(55.2)	29(61.7)	89(62.2)	362(60.9)	592(61.9)	
About average, n(%)	15(17.2)	12(25.5)	30(21.0)	120(20.2)	168(17.6)	
Higher than average, n(%)	24(27.6)	6(12.8)	24(16.8)	112(18.9)	197(20.6)	X^2^ = 8.66
***Age***, *M(SD)*	61.6(11.4)	68.4(10.5)	66.8(12.3)	66.1(11.3)	66.2(12.0)	F = 3.7**
**Health**						
***Health status***						
Poor health, n(%)	42(47.7)	13(27.7)	63(44.4)	313(52.9)	478(49.6)	
Good health, n(%)	46(52.3)	34(72.3)	79(55.6)	279(47.1)	485(50.4)	X^2^ = 13.27*
***Number of comorbidities***, M(SD)	1.7(1.3)	1.6(1.4)	1.9(1.4)	2.1(1.5)	1.9(1.4)	F = 2.6*
**Illness management characteristics**						
***Self-management***						
HEIQ (Skills), M(SD)	12.2(1.8)	11.6(1.7)	11.8(2.6)	11.6(2.18)	11.4(2.4)	K = 16.2**
HEIQ (Self-monitoring), M(SD)	19.5(2.6)	18.8(1.8)	18.9(3.4)	18.7(2.7)	18.5(2.9)	K = 11.2*
***Network work***						
Informational, M(SD)	4.5(3.1)	4.11(3.1)	4.19(3.5)	3.14(2.5)	2.03(2.2)	F = 51.14***
Practical, M(SD)	3.58(2.6)	3.60(2.8)	4.35(2.9)	2.66(1.7)	1.14(1.6)	F = 156.22***
Emotional, M(SD)	7.15(3.0)	7.40(3.1)	7.68(3.5)	4.52(2.3)	2.05(2.1)	F = 311.565***
***Well-being***						
*Happiness*, M(SD)	5.70(2.6)	6.66(2.6)	6.84(2.5)	6.10(2.4)	6.32(2.4)	F = 5.01***
Mental health, M(SD)	47.66(11.5)	53.20(10.1)	49.02(12.7)	46.13(12.1)	47.67(12.4)	F = 4.86**
**Health service utilisation**						
Number GP or nurse visits M(SD)	4.47(3.5)	3.91(3.7)	4.69(5.7)	5.08(4.0)	4.16(3.8)	K = 40.49***
A&E visits M(SD)	.26(.5)	.32(.8)	.16(.5)	.35(1.2)	.26(.9)	K = 9.21
Nights in hospital M(SD)	1.2(2.6)	.74(3.3)	.96(3.2)	1.49(5.5)	.95(4.5)	K = 11.96*
Number of feet examinations M(SD)	1.18(1.6)	3.62(14.3)	1.14(3.5)	1.26(3.7)	.96(2.0)	K = 16.63**

***p <.001,

**p <.01,

*p <.05

People in *diverse networks* were slightly more likely to be men than women, to have an advanced level of education and higher than average income. They were more likely than people in other network types to be employed. Information support from network members together with the reporting of good self-management skills and monitoring were highest here as well. However, the ‘egos’ also reported some of the lowest levels of well-being.

People located in *weak ties networks* were similar in most respects to those in the *diverse network*. However, unlike *diverse networks*, people with *weak ties networks* did not report high levels of self-management skills and monitoring. People with *weak ties networks* tended to be older, in relatively good health, and reported some of the highest level of well-being out of all the network types. *A* w*eak ties* network was the least cost intensive network type with cost primarily driven by low number of nights in hospital (see Table 3).

**Table 3:** Mean Cost of Healthcare resource utilisation (6 months) according to typology—(UK unit cost, €)*.

	GP visits	Hospital emergency room	Nights in hospital	Feet examination	Total cost
**Restricted**	320.67	49.71	549.60	43.82	963.81
**Minimal family**	392.19	286.80	849.90	56.48	1585.37
**Family**	359.89	28.68	572.27	54.67	1015.51
**Weak Ties**	300.68	61.18	419.28	163.55	944.70
**Diverse**	343.74	49.71	679.92	53.31	1126.69

* In order to calculate the overall cost of resource utilisation we explored number of GP visits, hospital emergency room visits, nights in hospital, number of feet examinations over the last 6 months and then used the UK unit cost for each service (PSSRU 2014) across the all network types.

*People in Family supported networks* were more likely to be women, and were characterised by lower education and income levels amongst ‘egos’, but had high levels of network member involvement and reported high levels of well-being.

Members of *minimal family networks* reported similarities to people with *family networks* in terms of education and income, but were in poorer health, receiving little support from network members, and reporting low well-being. The minimal family network was estimated to be the most costly in terms of healthcare utilisation with costs driven by inpatient length of stay and the use of A&E.

People with *restricted networks* had similar characteristics to people with minimal family networks, but, somewhat surprisingly, tended to have higher socio-economic status, be in better health, and report better well-being, although they also reported much lower levels of network member support (the lowest in the sample).

Restricted and minimal family networks, the two networks with the smallest number of network members, were at opposite ends in terms of level of healthcare utilisation. People with restricted networks had some of the lowest levels of utilisation of healthcare services, but given that people in such networks reported some of the highest levels of comorbidity and poor health, the low level of healthcare utilisation might indicate poor access to services or inability to get sufficient professional support when such support is needed. In contrast, the very high healthcare utilisation cost of people with minimal family networks might be due to the presence of a small number of social network members who can provide sufficient support to identify need and link people with CI to the healthcare services, but have insufficient capacity to offer illness management support.

It was the three network types which had a large number of network members (diverse, weak ties, and family) where healthcare utilisation was most likely to correspond to existing health needs. The healthcare utilisation cost in these networks was much lower than in limited family networks. This might in part be due to the extensive social network support that people with such networks can rely on in addition to the support of identifying CIM need and linking to healthcare services [6].

### Stage 3: Multivariate regressions analysis

Table 4 presents the estimated odds ratios for the adjusted effect of the explanatory variables on the outcome variables resulting from multivariable logistic regression analyses. The outcomes of self-management (skills and monitoring), network member work (informational, emotional, practical), and well-being (happiness and mental health) were regressed on network type, age, gender, number of network members, country of respondent, education, parents background, income, health status, and comorbidities.

**Table 4:** Network type, self-management, network work and well-being: Multivariable logistic regressions.

		Self-management				Network work						Well-being			
		*Skills*		*Self-monitoring*		*Information*		*Emotional*		*Practical*		*MCS (SF-12)*		*Happiness*	
*Variables*	*Categories*	OR	95% CI	OR	95% CI	OR	95% CI	OR	95% CI	OR	95% CI	OR	95% CI	OR	95% CI
**Network type**	*(Restricted)*														
	*Minimal family*	1.19	.93–1.53	1.56*	1.01–2.39	1.65***	1.28–2.13	9.22***	6.79–12.51	6.22***	4.61–8.38	.88	.67–1.15	1.00	.76–1.31
	*Family*	1.10	.70–1.73	.65	.31–1.40	1.18	.73–1.89	14.18***	7.00–28.75	14.00***	7.75–25.29	1.45	.89–2.38	1.57	.94–2.65
	*Weak ties*	1.40	.69–2.87	4.01	.53–31.42	1.49	.73–3.05	46.57***	10.27–211.12	7.40***	3.22–17.00	2.08	.89–4.89	.70	.32–1.56
	*Diverse*	1.98*	1.10–3.56	1.78	.49–6.4	1.07	.62–1.86	11.01***	4.14–29.34	4.44***	2.35–8.37	.91	.51–1.62	1.06	.58–1.95
**Age**	*(Up to 59)*														
	*60–74*	1.65***	1.27–2.14	1.15	.96–2.39	1.10	.84–1.5	1.22	.86–1.69	1.21	.90–1.63	1.77***	1.33–2.34	1.37*	1.03–1.82
	*75 and over*	1.01	.77–1.45	.95	.57–1.58	.98	.71–1.37	1.03	.70–1.52	1.09	.76–1.57	2.00***	1.42–2.83	1.28	.90–1.80
**Gender**	*(Men)*														
	*Women*	1.24	1.00–1.54	1.43	.99–2.06	.94	.74–1.18	1.35*	1.03–1.76	.44***	.34-.57	.75*	.60–100	.90	.71–1.15
**Country**	*(Bulgaria*)														
	*Greece*	1.52*	1.03–2.25	2.81**	1.51–5.23	.21***	.14-.33	.14***	.08-.22	.05***	.03-.08	.58*	.38-.90	2.41***	1.59–3.64
	*Netherlands*	3.11***	1.89–5.12	2.20	.97–5.00	2.16**	1.27–3.67	1.19	.84–3.92	.81	.40–1.65	3.75***	2.16–6.52	14.79***	8.30–26.35
	*Norway*	3.35***	2.30–4.89	3.32***	1.69–6.53	.61**	.42-.89	.32***	.20-.52	.08***	.05-.13	2.16***	1.46–3.20	.43***	.28-.66
	*Spain*	2.90***	1.89–4.45	2.88**	1.52–5.45	1.30	.84–2.01	.72	.43–1.21	.29***	.17-.50	2.81***	1.77–4.47	7.21***	4.54–11.44
	*United Kingdom*	3.63***	2.45–5.37	4.87***	2.35-10-12	1.22	.82–1.81	.31***	.19-.52	.21***	.13-.36	2.63***	1.77–3.98	6.38***	4.23–9.61
***Network members***	*(No network members)*														
	*1 to 3*	.89	.60–1.33	1.01	.54–2.14	5.80***	3.65–9.23	12.95***	6.42–26.14	14.56***	7.92–26.75	2.19**	1.39–3.44	1.23	.78–1.93
	*4 or more*	1.14	.70–1.86	1.76	.72–4.29	22.22***	12.68–38.95	53.49***	24.07–118.87	16.87***	8.55–33.30	1.74*	1.02–2.96	1.21	.71–2.06
**Education**	*(No education)*														
	*Primary school*	1.81	.96–3.42	1.97	.94–4.15	.77	.40–1.48	1.09	.51–2.35	.57	.26–1.25	1.21	.60–2.44	1.55	.82–2.92
	*Secondary school*	1.73	.88–3.41	2.28	.99–5.28	1.17	.59–2.34	1.58	.70–3.55	.78	.34–1.78	1.72	.81–3.64	1.96	.99–3.91
	*College*	2.45*	1.23–4.87	3.94**	1.64–9.46	1.24	.62–2.51	1.94	.85–4.45	.81	.35–1.88	2.21*	1.03–4.73	1.97	.98–3.97
	*University*	3.11**	1.51–6.40	9.38***	2.96–29.75	.92	.44–1.91	1.51	.64–3.60	.62	.26–1.49	1.65	.75–3.64	2.00	.96–4.18
**Parents**	(Born in country)														
	Not born in country	.87	.60–1.25	.68	.36–1.26	.64*	.43-.96	.58*	.37-.91	1.02	.68–1.52	.56**	.38-.83	.58**	.39-.87
**Income**	(Lower)														
	About average	1.18	.89–1.56	1.26	.76–2.10	.93	.69–1.26	1.21	.85–1.72	.69*	.50-.95	1.11	.81–1.16	1.32	.96–1.81
	Higher	1.57**	1.15–2.14	1.49	.83–2.66	1.11	.81–1.53	.85	.59–1.24	.79	.55–1.11	1.46*	1.05–2.04	1.35	.96–1.88
**Health**	(Poor health)														
	Good health	1.71***	1.38–2.14	1.90**	1.28–2.84	1.12	.88–1.41	1.00	.77–1.33	.79	.61–1.02	4.75***	3.75–6.02	2.80***	2.19–3.56
**Comorbidities**	(No comorbidities)														
	1 or 2	.77	.56–1.05	1.51	.88–2.58	.88	.64–1.21	.98	.67–1.43	1.11	.78–1.58	.97	.70–1.36	.75	.54–1.05
	3 or more	.87	.61–1.25	1.24	.69–2.22	1.20	.83–1.41	1.38	.89–2.12	1.16	.77–1.73	.71	.49–1.05	.68*	.46–1.00

***p <.001,

**p <.01,

*p <.05

The association between all network types and two of the outcome variables (support: emotional and practical) remained statistically significant after controlling for health status and socio-demographic factors. Furthermore, the association between network type and three other outcomes (self-management skills, self-monitoring, and information) was significant although the effects were weaker. Network type was not found to be significant for the other outcome measures (happiness and mental health).

Specifically, people who reported good self-management skills were more likely to have a *diverse* network, to be older, to be in relatively good health, to have high levels of income and education, and to live in the wealthier of the six countries (Norway, UK, Netherlands, Spain). High levels of self-monitoring were also associated with high education and relatively good health. Respondents with minimal family networks were more likely to report good self-monitoring compared to respondents with restricted networks. Country level differences were significant, with high levels of self-monitoring in the UK and Norway and low levels for Bulgarian respondents.

People who reported high levels of emotional support were more likely to have weak ties networks, to be women, to have native-born parents. Those who reported high levels of practical work were likely to have family networks, to be men, to have lower than average income. High levels of informational support were associated with minimal family network, and with parents being native born.

People who reported good mental health were more likely to have larger number of network members, have native born parents, to be older, to be men, have higher level of education, be in relatively good health, and to live in a wealthier country (Norway, UK, Netherlands, Spain).

## Discussion

On the basis of quantitative data using a constellation of network indicators (number, type and frequency of contact with network members) we have shown how it is possible to identify similarities and differences between sub-sets of personal networks and on this bases construct a typology related to people managing a LTC. The identified typology in this study offers further insights into the capacity of network connections to support LTCM and the identification of differing personal network types in this study suggests that some are more beneficial for promoting the support needed for living with and self-managing a long term condition, such as diabetes, than others. In this cross-sectional study, the observed correlations reflect both social dynamics in the networks and selection of network members over time.

The more restricted network types (e.g. restricted, minimal family) compared to the networks with larger number of ties (diverse, weak ties, and family) presented above show differences in the capacity to facilitate connections for LTCM and the type of support they receive from network members. Egos that had diverse networks were more likely than all other network types to report understanding of and skills in managing their long-term condition, while people in restricted networks were least likely to report such skills. Diverse networks were associated with service utilisation costs likely to correspond to healthcare need and better capacity to manage illness presumably due to the number and range of connections. More connections may lead to experiencing better health and well-being lessening the need to recourse to more formal services [28]. This is consistent with findings from other studies that have identified an association between social involvement and LTCM [6,21]. Social involvement is associated with opportunities for accessing a range of resources and support from a diverse set of ties [29]. Our previous research has shown that the provision of support work (emotional, practical, illness) is unlikely to be available from one type of relationship. It is spread across a range of types of network members with a limited degree of substitution for network members who are not present or disappear from a network over time [5,21].

The more restricted types of social support networks include a greater reliance on family members which can mean limited opportunities for accessing resources and a range of support. For example emotional and informational support provided by family members may be insufficient and complex to negotiate thus making support from weaker ties of acquaintances and friends an important addition to meeting the needs of people with LTCs [30]. While networks dominated by family members can provide supportive environments for people with LTCs this is likely to be contingent on the capacity and willingness of network members to negotiate existing roles and relationships, and the resources available within the network [21]. The substantial identity and emotional investment that people have in family ties makes their renegotiation a potentially difficult and time consuming process with interventions likely to only have limited impact. Research on social networks in a range of areas of social life shows how diverse and weak ties have a greater capacity to cut across the boundaries of more homogeneous networks, thus decreasing the constraints on access to resources [31,32]. In LTCM weak ties have in addition been associated with greater durability, less liability to loss over time than stronger ties and the enabling the moral positioning of the self-managing ‘self’ on the basis of a strong sense of reciprocity [33].

Over the longer term, diverse type networks are likely to be well suited to the on-going changes and contingencies associated with LTCM because a variety of roles and relationships are likely to offer flexibility and adaptability and opportunities for navigation, negotiation and harnessing of collective efficacy as necessary for leveraging support from a network in the context of a LTC [21]. Diverse as opposed to more restricted network types are likely to support LTCM by increasing individual capacity for negotiating change by offering respite from relationships where change might be problematic to negotiate (such as in close families), and by providing interactions that have the capacity to build further on individuals’ capabilities for managing and living with a LTC.

Whilst diversity results in greater opportunities and connections to support, this study found no association between diverse networks and well-being [26]. People in diverse type networks in this study reported lower well-being than people with the other types of networks indicating the existence of a tension between achieving improved LTCM and a sense of well-being. Whilst diverse networks may be better placed (compared to other network types) to support LTCM due to increased opportunities for negotiating relationships with network members, people in such networks may also have to deal with the burden of negotiating roles and responsibilities across relationship types, which may result in a negative impact on well-being as a result of emotional overload [34]. Additionally, diverse networks are likely to be more demanding on the time and effort expended by the person with a long term condition (compared to family orientated or restricted type networks) in terms of the capacity for reciprocation and retaining a sense of moral worth, in a context of experiencing physical incapacity. The burden of negotiation in maintaining commitment and connectivity to numerous and diverse network members may limit capacity to feel equal and of value to others. This might lead to relatively lower subjective well-being gain from relationships than those inhabiting more restricted networks [35]. The availability of a diverse set of local organisations with a variety of logics of interpersonal responsibility and reciprocity is likely to increase the likelihood of building diverse networks that are well suited to people’s preferences and circumstances and with low burden of relationship negotiation [36,37,38].

### Policy implications

In our analysis restricted or minimal family networks constituted the two largest groups, however, whilst uncommon, diverse network types are present and show potential benefits for people with long-term conditions, such as diabetes. In this respect our findings offer a clear direction for policy through support for extending and deepening engagement with existing network members in all network types and by enhancing diverse networks as the optimal network type for LTCM. Adopting such a policy focus is possible in practice given that recent studies have demonstrated that social network interventions can be effective in changing the structure of the networks of people with LTCs [39]. However, network interventions are most likely to be effective if they take into consideration the burden of negotiating relationships, and need to be tailored to individual preferences and capabilities [35].

### Limitations and future work

It should be noted that, recruitment for the study was from predominantly deprived and marginalised settings as these are contexts where CIM is likely to be most challenging. However, this recruitment focus makes it likely that networks with a smaller number of network members and lower level of support are overrepresented in our sample than in the general population. Additionally, these findings need to be interpreted with caution due to the method used and because findings vary between countries. K-means clustering which has been used in order to derive the network types has drawbacks in that it is both deterministic (i.e. driven by the data itself) and sensitive to changes in the characterising variables used to feed the algorithm. Given the large variation in the level of service utilisation across countries and the organisation of the health service systems in each country the type of network is unlikely to be associated with service use in a simple way. Further work will need to explore these variations, but the wider environments (e.g. type of welfare system, organisation of healthcare provision) are likely to shape the type of network engagement with illness management support in different countries. Additionally, further work may also need to be directed towards developing more sensitive network typologies that would capture diversity within family dominated networks.
